# Screening for negative emotions and analysis of related factors among general surgery inpatients: a retrospective cross-sectional study

**DOI:** 10.3389/fpsyg.2024.1343164

**Published:** 2024-02-06

**Authors:** Jie Yang, Qingyun Xie, Bing Chen, Jun Wang, Lijun Wang, Chuying Luo, Yang Zhang, Hui Xiong, Qingqing Xiang, Zehua Lei, Guojun Zeng

**Affiliations:** Department of Hepato-Pancreato-Biliary Surgery, The People’s Hospital of Leshan, Leshan, China

**Keywords:** anxiety, depression, psychological screening, general surgery, COVID-19 pandemic

## Abstract

**Background:**

Adverse psychological states in surgical patients can impact outcomes. This study aimed to evaluate mood disorders and associated factors in general surgery inpatients using the Huaxi Emotional Distress Index (HEI).

**Methods:**

This retrospective cross-sectional study analyzed HEI scores of 20,398 adult patients hospitalized for elective surgery at a tertiary hospital in China (2018–2021). Univariable and multivariable logistic regression identified factors linked to moderate/severe mood disturbances.

**Results:**

Factors linked to moderate/severe mood disturbances were identified through univariable and multivariable logistic regression. The results showed that 3.7% of the patients had HEI ≥ 13, indicating significant emotional issues. The mean age was 52.67 (16.14) years in the group with no/mild distress and 59.65 (16.34) years in the group with moderate/severe distress. Among all the cases included, there were 2,689 cases (13.18%) of gastric and esophageal diseases, 1,437 cases (7.04%) of hepatic diseases, 913 cases (4.47%) of periampullary and pancreatic tumors, 9,150 cases (44.85%) of gallbladder diseases, 2,777 cases (13.61%) of colorectal diseases, and 3,432 cases (16.83%) of other diseases. The male percentage was 45.5 and 54.9% in the two groups, respectively. Older age, male gender, unstable occupations, lower education, and unmarried status were associated with higher risks of mood disturbances (all *p* < 0.05). A significant downward trend in adverse emotions was observed with increasing education levels (*p* < 0.001). Furthermore, the study found that the inpatients had higher HEI scores prior to the COVID-19 pandemic compared to during the pandemic (*p* < 0.001). However, the occurrence of adverse mood states in these patients was not exacerbated by the COVID-19 pandemic. The trend test remained highly significant in the none-adjusted, age-sex adjusted, and fully adjusted models (all *p* for trend <0.001).

**Conclusion:**

The implementation of routine screening in higher education institutions (HEIs) allows for the early identification of surgical inpatients who require psychological intervention. It is recommended that counseling services focus on individuals with lower levels of education and income instability in order to address negative mood states. Furthermore, the potential application of this screening system in other clinical settings could enable earlier psychological interventions for a larger number of patients.

## Introduction

For the majority of patients, illness, hospitalization, and surgery are significant events that can result in different levels of anxiety, depression, and other negative emotions. If severe or long-lasting, these conditions can affect patients’ physical and psychological well-being, hinder recovery, worsen existing illnesses, and potentially give rise to new psychological problems (Yilmaz et al., 2012). Anxiety is a prevalent psychological response observed in patients awaiting different surgical procedures, with reported incidence rates ranging from 11 to 80% in preoperative assessments of adult patients (Zemła et al., 2019; Wang et al., 2022). Preoperative anxiety can have various effects, including influencing postoperative pain levels, anesthesia requirements, and the need for analgesics. Recent findings shown the preoperative stress (anxiety, lack of sleep, fasting) may have been associated with alterations of the commensal microflora and may contribute to increased postoperative complications and mortality rates (Lukovic et al., 2019). Accurately screening for patients with pre-existing adverse mood states upon admission and implementing appropriate psychological interventions for these high-risk groups is crucial. Our objective was to examine the distribution of mood disorders among these hospitalized patients and identify relevant influencing factors. The findings of this cross-sectional study will contribute to the development of effective psychological counseling strategies for inpatients in the future.

## Methods

This study was approved by the Ethics Committee of People’s Hospital of Leshan and was performed in accordance with the Declaration of Helsinki. All data in this study were extracted from the hospital’s electronic medical record system. All subjects provided written informed consent.

### Patients

In this retrospective study, we collected basic information and administered the ‘Huaxi Emotional Distress Index (HEI)’ (Wang et al., 2017) questionnaire to 20,398 general surgery inpatients at our hospital between December 2018 and December 2021. To be included in the study, participants had to meet certain criteria. They needed to be aged 18 or older, hospitalized for elective surgery, and have complete medical records with no missing data. Patients who underwent emergency surgery, were unable to complete the questionnaire independently, were in a comatose state, or had incomplete medical records or missing data were excluded. Additionally, individuals under 18, those with impaired cognitive function, previous diagnosis of mental illness, administration of psychiatric medications, or intellectual disability were also excluded. By focusing on adult elective surgery patients with intact cognitive function and complete data, the study population represents typical surgical inpatients suitable for an initial investigation of mood disorders in this setting. The clearly defined eligibility criteria ensure that the included patients are representative of the target population for whom preoperative psychological screening is most relevant in real-world clinical practice.

### Investigation method and tool

#### Method

Prior to assessment, nurses should briefly introduce the necessity and significance of completing the questionnaire to patients using the following standardized language:

“Hello, I’m your nurse ___. To provide you with better service, we need to understand your recent psychological health status. Please fill out this questionnaire according to your conditions in the past month honestly and independently. Your genuine responses will facilitate us to offer you better diagnosis and treatment. Your questionnaire answers will be kept confidential.”

The assessment should avoid influence from the patient’s family members or companions. If needed, nurses can clearly and accurately read out the items and options to patients, avoiding explaining items based on personal understanding. If patients have questions, nurses should instruct them to respond based on their own understanding. After completion, nurses should promptly check the integrity of the questionnaires.

#### Tool

The HEI scale (Wang et al., 2017), developed by the Mental Health Center of West China Hospital, was used as an emotional screening tool in this study. It assesses patients’ mood and psychological state over the past month to quickly identify those with adverse emotions (depression and/or anxiety) and related mental health issues, as well as evaluate their severity. The questionnaire used in this study consists of four dimensions and nine items, which include depressive symptoms (items 1, 2, 7, 8), anxiety symptoms (items 3, 4, 5), acute anxiety (item 6), and suicide-related (item 9). Each item is scored on a 5-point Likert scale, ranging from ‘none of the time’ (0) to ‘all of the time’ (4). The total scores range from 0 to 36. The validity of the scale is 0.898, indicating good test–retest reliability over time. The Cronbach’s alpha coefficient for internal consistency is 0.90, and the factor loadings for the items range from 0.695 to 0.801, suggesting excellent reliability and validity.

All patients underwent psychological assessment using the HEI questionnaire on the day of admission. The questionnaire utilizes a 5-point Likert scale, with scores ranging from 0 to 4. Higher scores indicate a greater impact. Total scores below 9 indicate no adverse emotions, scores between 9 and 12 indicate mild adverse emotions without the need for clinical intervention, scores between 13 and 16 indicate moderate adverse emotions, and scores equal to or higher than 17 indicate relatively severe or severe adverse emotions, with these patients being assessed as being at risk of suicide and requiring clinical intervention. Furthermore, for patients scoring 2 or higher on item 9, after verifying the accuracy of the score, further systematic suicide risk assessment should be performed. For those identified with severe psychological issues based on total scores, systematic suicide risk assessment should be conducted regardless of the score on item 9. Patients with medium to high suicide risk require 24-h accompaniment by family members or companions, and psychiatric consultation if necessary.

### Statistical analysis

For parameters with continuous data, the normal distribution was expressed as mean ± standard deviation, and the skewed distribution was expressed as median (M) and quartile range (P25 − P75). Count data were expressed as frequencies and percentage (%). Normality was established using Kolmogorov Smirnov test. Comparison of groups was performed using Kruskal-Wallis test for non-normality continuous variables. Patients’ education levels were grouped into three categories: low (illiterate or completed elementary school), middle (up to high school), and high (college or university). To investigate the associations between demographic characteristics and severe adverse emotions, we used univariable and multivariable logistic regression analysis, including gender, age, occupation category, education level, marital status, “before and during the COVID-19 pandemic” and ethnicity as independent variables. Using the Cochran-Armitage test, the trend in adverse emotions was examined across all educational levels. Statistical analyses were performed using R software version 4.0.3 (The R Foundation, Vienna, Austria) and EasyR software.^1^ All tests were two-sided. *p* < 0.05 was considered significant.

## Results

### Distribution of HEI assessment score

The study included 20,398 patients, 19,645 had HEI scores ≤13, indicating no or mild adverse emotions that did not require psychological intervention. On the other hand, 753 patients had HEI scores ≥14, indicating moderate to severe adverse emotions. Of these, 2.1% (422 cases) had moderate adverse emotions, while 1.6% (331 cases) had severe adverse emotions (Table 1). These patients were assessed to be at risk of suicide. However, after receiving psychological counseling and care from psychosomatic medicine and ward staff, no serious consequences such as suicide due to adverse emotions were observed.

**Table 1:** Distribution of demographic characteristics.

HEI assessment score	Number of patients (*n*)	Percentage (%)
0–8 points: no adverse emotions	18,178	89.1
9–12 points: mild adverse emotions	1,467	7.2
13–16 points: moderate adverse emotions	422	2.1
17–36 points: severe adverse emotions	331	1.6

HEI: Huaxi emotional intelligence index.

### Epidemiological characteristics

Among the included patients, there was no significant difference in ethnicity (*p* = 0.334) and type of operation (*p* = 0.425). The mean age was 52.67 years (*SD* = 16.14) in the “None and Mild Adverse Emotions (HEI score < 13) “group and 59.65 years (*SD* = 16.34) in the “Moderate and Severe Adverse Emotions (HEI score≧13)” group (*p* < 0.001). There was a significant difference in the gender distribution, with 45.47% males in the former group and 54.85% in the latter group (*p* < 0.001). Occupation (*p* < 0.05) and marital status (*p* = 0.001) also showed significant differences. Education levels had notable disparities between the two groups. In the “None and Mild Adverse Emotions” group, 40.17% had low education, 44.20% had a middle education, and 15.63% had a high education. Conversely, in the “Moderate and Severe Adverse Emotions” group, a higher proportion had low education (54.65%), while fewer had a middle (36.17%) or high education (9.18%) (all *p* < 0.001) (as shown in Table 2).

**Table 2:** Epidemiological characteristics.

	HEI score < 13 (*n* = 19,645)	HEI score ≥ 13 (*n* = 753)	*P*
Age ($\bar{x}$±s, years)	52.67 (16.14)	59.65 (16.34)	<0.001
Gender, *n* (%)			<0.001
Male	8,932 (45.47%)	413 (54.85%)	
Female	10,711 (54.53%)	340 (45.15%)	
Occupation category, *n* (%)			0.049
Regular income	4,001 (20.37%)	180 (23.90%)	
Irregular income	8,907 (45.36%)	318 (42.23%)	
Others	6,730 (34.27%)	255 (33.86%)	
Education level, *n* (%)			<0.001
7 pt	7,877 (40.17%)	411 (54.65%)	
Middle	8,669 (44.20%)	272 (36.17%)	
High	3,065 (15.63%)	69 (9.18%)	
Ethnic, *n* (%)			0.334
Han	19,143 (97.44%)	738 (98.01%)	
Others	502 (2.56%)	15 (1.99%)	
Marital status, *n* (%)			0.001
Unmarried	1,030 (5.24%)	23 (3.05%)	
Married	18,177 (92.55%)	723 (96.02%)	
Others	433 (2.20%)	7 (0.93%)	
COVID-19, *n* (%)			<0.001
Before	8,292 (42.21%)	418 (55.51%)	
During	11,353 (57.79%)	335 (44.49%)	
Type of operation, *n* (%)			0.425
Gastroesophageal surgery	2,584 (13.11%)	105 (13.94%)	
Hepatectomy	1,392 (7.09%)	45 (5.97%)	
Pancreatectomy	879 (4.47%)	34 (4.65%)	
Cholecystectomy	8,839 (45.02%)	311 (41.17%)	
Colorectal resection	2,667 (13.58%)	110 (14.61%)	
Other surgery	3,284 (16.71%)	148 (19.66%)	

### Univariate logistic regression and multivariate logistic regression

In the regression analysis, the ‘HEI scores level’ was used as the dependent variable. The results presented in Table 3 indicate that several factors were associated with lower odds of experiencing adverse emotions in the univariable analysis. These factors include being female (*OR* = 0.69, *p* < 0.001), younger age (*OR* = 1.03, *p* < 0.001), having irregular income (*OR* = 0.79, *p* = 0.015), and having higher education (*OR* = 0.60 and 0.43 for middle and high, respectively, both *p* < 0.001). Marital status and the impact of COVID-19 were also significant predictors. These associations remained significant in the multivariable analysis, highlighting the independent contributions of gender, age, occupation, education, marital status, and COVID-19 impact to emotional well-being. However, ethnicity did not show a significant association in either analysis.

**Table 3:** Univariable and multivariable analysis.

	Univariable analysis	Multivariable analysis
	*OR* (95%CI) *p*-value	*OR* (95%CI) *p*-value
Gender, *n* (%)		
Male	Ref	Ref
Female	0.69 (0.59, 0.79) <0.001	0.72 (0.62,0.84) <0.001
Age ($\bar{x}$±s, years)	1.03 (1.02, 1.03) <0.001	1.02 (1.02,1.03) <0.001
Occupation category, *n* (%)		
Regular income	Ref	Ref
Irregular income	0.79 (0.66, 0.96) 0.015	0.70 (0.57,0.85) <0.001
Others	0.84 (0.69, 1.02) 0.084	0.68 (0.56,0.84) <0.001
Education level, *n* (%)		
Low	Ref	Ref
Middle	0.60 (0.51, 0.70) <0.001	0.70 (0.59,0.83) <0.001
High	0.43 (0.33, 0.56) <0.001	0.60 (0.44,0.81) <0.001
Marital status, *n* (%)		
Unmarried	Ref	Ref
Married	1.78 (1.17, 2.71) 0.007	0.83 (0.53,1.29) 0.408
Others	0.72 (0.31, 1.70) 0.458	0.31 (0.13,0.73) 0.007
COVID-19, *n* (%)		
Before	Ref	Ref
During	0.59 (0.51, 0.68) <0.001	0.56 (0.48,0.65) <0.001
Gender, *n* (%)		
Male	Ref	Ref
Female	0.78 (0.46, 1.30) 0.336	0.87 (0.50,1.49) 0.603

### Trend test FOE education

A significant decreasing trend in the risk of adverse emotions was observed among individuals with higher education levels. This trend remained highly significant in the none-adjusted, age-sex adjusted, and fully adjusted models (all *p* for trend <0.001), indicating a consistent association between higher education and a reduced likelihood of experiencing adverse emotions, as presented in Table 4.

**Table 4:** Trend test FOE education.

Education	None-adjusted	Age-sex adjusted	Fully adjusted
Low	Ref	Ref	Ref
Middle	0.60 (0.51, 0.70) <0.0001	0.75 (0.63, 0.88) 0.0006	0.71 (0.59, 0.84) <0.0001
High	0.43 (0.33, 0.56) <0.0001	0.69 (0.52, 0.91) 0.0099	0.63 (0.46, 0.86) 0.0030
*P* for trend	<0.001	<0.001	<0.001

## Discussion

Adverse mood states have received significant attention in recent years as a crucial factor that affects postoperative outcomes in surgical patients. These mood disturbances, which arise as a result of illness, hospitalization, and elective surgery, are collectively referred to as preoperative anxiety (Munafò and Stevenson, 2001). The level of severity of this preoperative anxiety has an impact on patients’ adherence to postoperative treatment plans, wound healing, and the occurrence of postoperative complications such as nausea, vomiting, and pain (Linn et al., 1988; Boeke et al., 1992). In China, there is a lack of awareness regarding the importance of preoperative anxiety in non-psychiatric clinical settings, particularly in surgical fields. As a result, interventions for preoperative anxiety are still relatively limited (Wang et al., 2017). To address this issue, the HEI has been developed as a tool for preliminary screening of adverse mood states such as depression, anxiety, and suicide risk among inpatients. Research conducted by Wang et al. (2023) has demonstrated the effectiveness of this questionnaire in hospitalized patients. While the HEI has its advantages in terms of simplicity, understandability, and efficiency, it poses challenges when used with patients who have lower educational levels or are illiterate. These patients may struggle to comprehend the questionnaire content and may require assistance from a healthcare provider to understand it. In some cases, patients may even provide inaccurate responses in order to hide their true condition, which could potentially affect the reliability and validity of the questionnaire.

The results from both univariate and multivariate analyses revealed that patients with different occupational attributes had significantly varying risks of moderate to severe adverse mood states, which required intervention. Patients employed in enterprises and institutions in China, such as civil servants and corporate employees, exhibited lower risks compared to self-employed individuals and farmers who were not employed in enterprises or institutions (*OR* = 0.79, 95%*CI* = 0.66–0.96, *p* = 0.0152). In China, these groups without stable jobs often experience income instability and relatively lower income levels. Moreover, the lack of steady work sometimes leads to inadequate health insurance coverage. Hospitalization and illness frequently impact their regular income and increase out-of-pocket medical expenses. Previous studies by Bauer et al. (2011) and Lemogne et al. (2019) have demonstrated that income level and socioeconomic status are associated with the likelihood of anxiety disorders in populations, with relatively low-income groups having significantly higher risks of mood disturbances. Hinata et al.’s (2021) study revealed a negative correlation between income and depression, suggesting that higher income levels are associated with lower incidence of depression. Similarly, Yang et al.’s (2020) found a positive correlation between the lack of social support and the occurrence of emotional disorders. Interestingly, stable employment was identified as a factor that contributes to better social support. This finding may help explain the observed differences in risks among different occupations in our study.

The cross-sectional survey study was conducted before and after the COVID-19 outbreak, and COVID-19 was included as a variable in the study. The analysis revealed that patients had a higher likelihood of experiencing moderate to severe adverse mood states prior to the COVID-19 pandemic compared to during the pandemic (*OR* = 0.59, 95%*CI* = 0.51–0.68, *p* < 0.001). A survey conducted during the COVID-19 pandemic in China examined the mental health status of the general population. The survey (Shi et al., 2020) included 56,679 individuals from 34 provinces nationwide. The findings revealed that the prevalence of depression was 27.9%, anxiety was 31.6%, insomnia was 29.2%, and acute stress symptoms were reported by 24.4% of the participants. Interestingly, despite the increased psychological pressure caused by the pandemic, the survey discovered that the HEI scores of patients admitted to the hospital after the pandemic were lower than those before. The survey revealed that the Hospital Experience Index (HEI) scores of patients admitted to the hospital after the pandemic were lower compared to those before. Despite no variations in the types of diseases and surgical procedures performed on admitted patients before and after the pandemic, we hypothesize that this decrease could be attributed to the implementation of strict ward management protocols during the pandemic. These protocols included restricted entry/exit, allowing only one accompanying family member per patient, and regular nucleic acid testing. These measures aimed to provide hospitalized patients with an increased sense of safety within the wards. Furthermore, studies have indicated that Chinese individuals are more likely to experience increased anxiety due to dissatisfaction with COVID-19 control measures (Shi et al., 2021). In tertiary general hospitals across China, strict ward management protocols were implemented during the pandemic. These protocols required admitted patients to provide negative nucleic acid test results and enforced closed-off management with limited caregivers. All personnel entering the wards were subjected to regular nucleic acid testing, ensuring that everyone inside the wards tested negative. As a result, the hospital environment was perceived as safer compared to the potential outbreaks that could occur outside the hospital, providing psychological reassurance.

In this study, we conducted a test to examine the relationship between education level and adverse mood states. The results revealed that individuals with lower education levels had higher probabilities of experiencing moderate and severe adverse mood states (all *p* for trend <0.001) and were more likely to require clinical intervention for their mood issues. Additionally, in a meta-analysis conducted by Walker et al. (2021), a total of 40 studies spanning a 15-year period were included. The survey data from 9,195 cancer patients from various Low- and Lower-Middle-Income Countries were analyzed. The findings revealed a strong association between depressive and anxiety symptoms with advanced disease and low levels of education. Chen et al.’s (2022) involved 966 elderly patients who were receiving trauma treatment at an orthopedic center. The study findings indicated that orthopedic trauma patients with higher education levels had a reduced risk of experiencing anxiety and depression compared to patients who were illiterate. Additionally, the study suggested that improved access to education and skills training can improve employment opportunities, leading to a stronger economic foundation and social support system. As a result, individuals are better prepared to handle setbacks and challenges due to their improved economic capacity. From a psychological standpoint, individuals with higher education are generally considered to possess better cognitive abilities. Cognitive theory suggests that cognitive processes influence emotions and behaviors (Bauer et al., 2011; Patria, 2022). When facing hospitalization and treatment for illness, individuals with lower education levels often lack accurate and comprehensive understanding of the disease itself, its treatment, and prognosis compared to those with higher education. Moreover, their means of acquiring knowledge may be less effective than those with higher education. Consequently, individuals with lower education levels have a higher likelihood of developing adverse mood states.

Preoperative anxiety can exacerbate postoperative pain and prolong recovery time. Preventing and alleviating preoperative anxiety has positive effects on patients’ overall psychological health (Friedrich et al., 2022), and patients with higher levels of preoperative anxiety also have greater needs for disease-related information (Perks et al., 2009). Our study found that factors including patients’ gender, age, occupation, education, marital status, and COVID-19 were correlated with moderate to severe adverse mood states in patients. Especially, patients’ education levels as well as the stability of their occupations and income warrant more attention. These results suggest that assessment of adverse mood states in patients should be strengthened during hospital admission to accurately identify high-risk groups, and detailed, personalized psychological counseling strategies that meet patients’ needs should be developed focusing on these high-risk factors and populations. Intervention measures should be implemented for early intervention, providing more information needed by patients, enhancing health guidance, alleviating their adverse moods, and avoiding adverse impacts on patients’ postoperative outcomes.

This retrospective study conducted at a single center has some limitations, such as a modest sample size and reliance on subjective survey data. These limitations reduce the generalizability of the findings and the ability to establish causality. To further validate these findings across diverse surgical populations, it would be beneficial to conduct large multi-center prospective studies using standardized scales. Additionally, comparing psychological interventions through randomized controlled trials could help determine the most effective approaches. Long-term follow-up studies would provide insights into whether preoperative screening improves postoperative outcomes. Further research is also needed to explore nurses’ training in emotional screening, the development of individualized care plans, and cost–benefit analysis to support the implementation of screening into clinical practice. While this study offers initial insights, it highlights the need for more rigorous research to validate and implement systematic preoperative mood screening.

## Conclusion

This study revealed a significant prevalence of moderate to severe anxiety and depression among general surgery inpatients. The utilization of the HEI scale for routine screening enables early detection of patients who are at risk of experiencing these adverse psychological states. Noteworthy factors associated with a higher likelihood of mood disorders included older age, male gender, irregular income source, lower education level, unmarried status, and previous hospitalization prior to the COVID-19 pandemic. It is crucial to focus on these high-risk groups and provide appropriate counseling and interventions to alleviate negative emotions before surgery, prevent unfavorable postoperative outcomes, and enhance recovery.

## Data availability statement

The original contributions presented in the study are included in the article/supplementary materials, further inquiries can be directed to the corresponding author/s.

## Ethics statement

The studies involving humans were approved by the Ethics Committee of People’s Hospital of Leshan. The studies were conducted in accordance with the local legislation and institutional requirements. Written informed consent for participation in this study was provided by the participants’ legal guardians/next of kin.

## Author contributions

JY: Writing – original draft, Conceptualization, Methodology, Project administration, Resources, Software, Writing – review & editing. QYX: Writing – original draft, Conceptualization, Data curation, Formal analysis, Investigation. BC: Data curation, Resources, Writing – review & editing. JW: Funding acquisition, Supervision, Writing – original draft. LW: Writing – review & editing, Software. CL: Writing – review & editing, Data curation, Visualization. YZ: Data curation, Funding acquisition, Writing – original draft. HX: Data curation, Writing – original draft, Conceptualization. QQX: Formal analysis, Writing – original draft. ZL: Conceptualization, Data curation, Formal analysis, Funding acquisition, Writing – review & editing. GZ: Data curation, Formal analysis, Writing – original draft, Writing – review & editing.

## Footnotes

^1^ https://www.easyr.cc
