# Supplementary material for: Screening for negative emotions and analysis of related factors among general surgery inpatients: a retrospective cross-sectional study
Source: Front Psychol. 2024 Feb 6;15:1343164. doi: 10.3389/fpsyg.2024.1343164 (PMC10876801; doi:10.3389/fpsyg.2024.1343164)
Supplement: Supplementary file 1 [file Table_1.docx]

**Supplementary Table**

Model ROC curve and best threshold analysis

| Model | AUC（95%CI） | Specificity | sensitivity |
| --- | --- | --- | --- |
| Full model | 0.8034（0.7871-0.8198） | 0.7186 | 0.7766 |


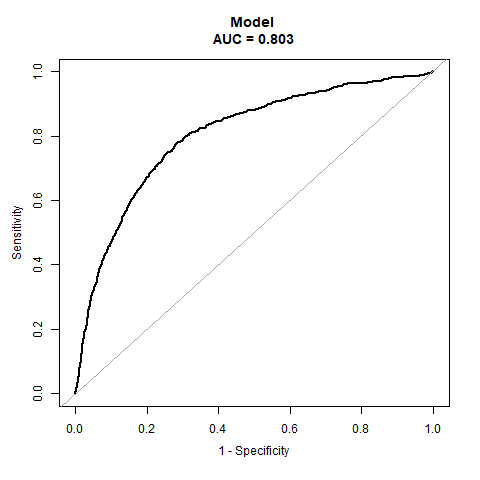


The ordinal outcome variable：HEI score

We included all variables from the multivariable analysis in Table 2.
